# Effect of Nanoparticle Size in Pt/SiO_2_ Catalyzed Nitrate Reduction in Liquid Phase

**DOI:** 10.3390/nano11010195

**Published:** 2021-01-14

**Authors:** Khawer Shafqat, Satu Pitkäaho, Minna Tiainen, Lenka Matějová, Riitta L. Keiski

**Affiliations:** 1 Environmental and Chemical Engineering Research Unit, University of Oulu, P.O. Box 4300, FI-90014 Oulu, Finland; khawer.shafqat@oulu.fi (K.S.); minna.tiainen@oulu.fi (M.T.); 2 Institute of Environmental Technology, CEET, VSB-Technical University of Ostrava, 17. Listopadu 2172/15, 708 00 Ostrava-Poruba, Czech Republic; lenka.matejova@vsb.cz

**Keywords:** nanoparticles, Pt, platinum, nitrate reduction, hydrogenation, wastewater, water treatment, environmental catalysis

## Abstract

Effect of platinum nanoparticle size on catalytic reduction of nitrate in liquid phase was examined under ambient conditions by using hydrogen as a reducing agent. For the size effect study, Pt nanoparticles with sizes of 2, 4 and 8 nm were loaded silica support. TEM images of Pt nanoparticles showed that homogeneous morphologies as well as narrow size distributions were achieved during the preparation. All three catalysts showed high activity and were able to reduce nitrate below the recommended limit of 50 mg/L in drinking water. The highest catalytic activity was seen with 8 nm platinum; however, the product selectivity for N_2_ was highest with 4 nm platinum. In addition, the possibility of PVP capping agent acting as a promoter in the reaction is highlighted.

## 1. Introduction

Platinum (Pt) is an important catalyst in environmental applications. The recent advances in nanoparticle synthesis offer new possibilities for utilizing this precious metal even more atom-efficiently in the future [1]. Lowering the metal loading by using single atoms as catalytically active sites is an appealing strategy. It is known that adjusting platinum size and shape can significantly influence the activity and especially the selectivity of structure sensitive reactions [2,3]. Changes in platinum nanoparticle size can cause relative shift in the d-band centers with respect to the fermi energy level, and, therefore, particle size in the nanoscale has a significant role to play in catalytic processes [4,5,6]. Platinum catalysts are considered very effective in various hydrogenation reactions for fundamental research and industrial applications [7]; however, most studies are carried out in gas phase [3]. It is worth examining the size effect of platinum nanoparticles at the solid–liquid interface, and therefore we chose to study nitrate (${\mathrm{NO}}_{3}^{-}$) reduction in aqueous phase.

Much is known about nitrate (${\mathrm{NO}}_{3}^{-}$) pollution of surface waters becoming a global concern due to the excessive use of fertilizers and discharge of nitrate-containing municipal and industrial wastewaters to the neighboring environment [8,9]. As a result, nitrate contamination of water bodies plays a crucial role in eutrophication process, posing a major threat to many surface waters bodies [10]. Elevated nitrate concentrations in drinking water can cause health problems among the public such as blue baby syndrome and stomach cancer [11,12]. In addition, high levels of nitrates in water can adversely affect marine animals, and, in some cases, acute nitrate poisoning in cattle has been reported [13]. Due to the adverse health effects, the World Health Organization (WHO) has proposed a limit of 50 mg/L nitrate (${\mathrm{NO}}_{3}^{-}$) in drinking water. Therefore, various physicochemical and biological treatment methods, such as adsorption [14,15,16], ion-exchange [17], reverse osmosis [18] and biological methods [19], have been used for nitrate removal from water. However, due to high solubility of nitrate in water, these methods are prone to several limitations, such as secondary waste generation, low removal efficiency, biofouling and high cost. As an alternative to conventional techniques, catalytic reduction of nitrate has been studied to some extent. In the late 1980s, pioneering work by Vorlop and coworkers [20] suggested a nitrate (${\mathrm{NO}}_{3}^{-}$) reduction method based on the use of a bimetallic catalyst and hydrogen gas as the reductant. Since then, most studies are focused on bimetallic catalytic systems, as it is assumed that catalytic nitrate reduction is occurring in two steps: first the reduction of nitrate (${\mathrm{NO}}_{3}^{-}$) to nitrite (${\mathrm{NO}}_{2}^{-}$) and then nitrite (${\mathrm{NO}}_{2}^{-}$) to nitrogen ($N_{2}$) and/or to ammonium (${\mathrm{NH}}_{4}^{+}$) [21,22,23]. Some studies have reported nitrate (${\mathrm{NO}}_{3}^{-}$) removal by using monometallic catalysts, but either the removal efficiencies were very low or the selectivity was higher towards ammonium (${\mathrm{NH}}_{4}^{+}$) as a byproduct [24,25]. The influence of Pt nanoparticle size on the catalyst performance has been observed in various hydrogenation reactions [26,27,28], but it should be noted that to our knowledge nitrate (${\mathrm{NO}}_{3}^{-}$) hydrogenation in liquid phase by using size-controlled supported Pt monometallic catalyst has not been studied yet. These facts indicate that there still exists a lack of knowledge how to make/prepare a monometallic catalyst to be an efficient and cheaper catalyst (compared to bimetallic catalysts) for the reduction of nitrate (${\mathrm{NO}}_{3}^{-}$).

Thus, in this study, platinum nanoparticles (NPs) with average sizes of 2, 4 and 8 nm were synthesized by a polyol method with Poly(vinylpyrrolidone) (PVP) as the capping agent. To study the size effect of Pt NPs properly, the NPs were incorporated into a high-surface-area 3D support. Mesoporous silica (SBA-15) and mesocelluous silica (MCF-17) were chosen as the catalyst supports and 2 and 4 nm Pt NPs were loaded on SBA-15 (average pore size ~7.4 nm), while 8 nm Pt NPs were loaded on MCF-17 (average pore size ~40 nm). The effect of nanoparticle size on catalytic activity was studied in nitrate reduction in an aqueous phase.

## 2. Materials and Methods

### 2.1. Catalyst Preparation

During the catalyst syntheses, water purified with a Milli-Q Advantage A10 system and analytical grade reagents and solvents were used. Poly(vinylpyrrolidone), platinum (II) acetylacetonate (~0.2 mmol), sodium borohydride (NaBH_4_, 98.0%), ethylene glycol (anhydrous, 99.8%) and hexane (anhydrous, 95.0%), triblock copolymer Pluronic P123, tetraethoxysilane (TEOS), 1,3,5-trimethylbenzene (TMB), hydrochloric acid (HCl) and chloroplatinic acid hexahydrate (~0.2 mmol) were purchased from Sigma-Aldrich (Espoo, Finland). Acetone and ethanol (96%) together with sodium nitrate (NaNO_3_, 99.0%) to prepare the stock solution of nitrate (${\mathrm{NO}}_{3}^{-}$) as a substrate were purchased from VWR International (Helsinki, Finland).

**Figure 1:**
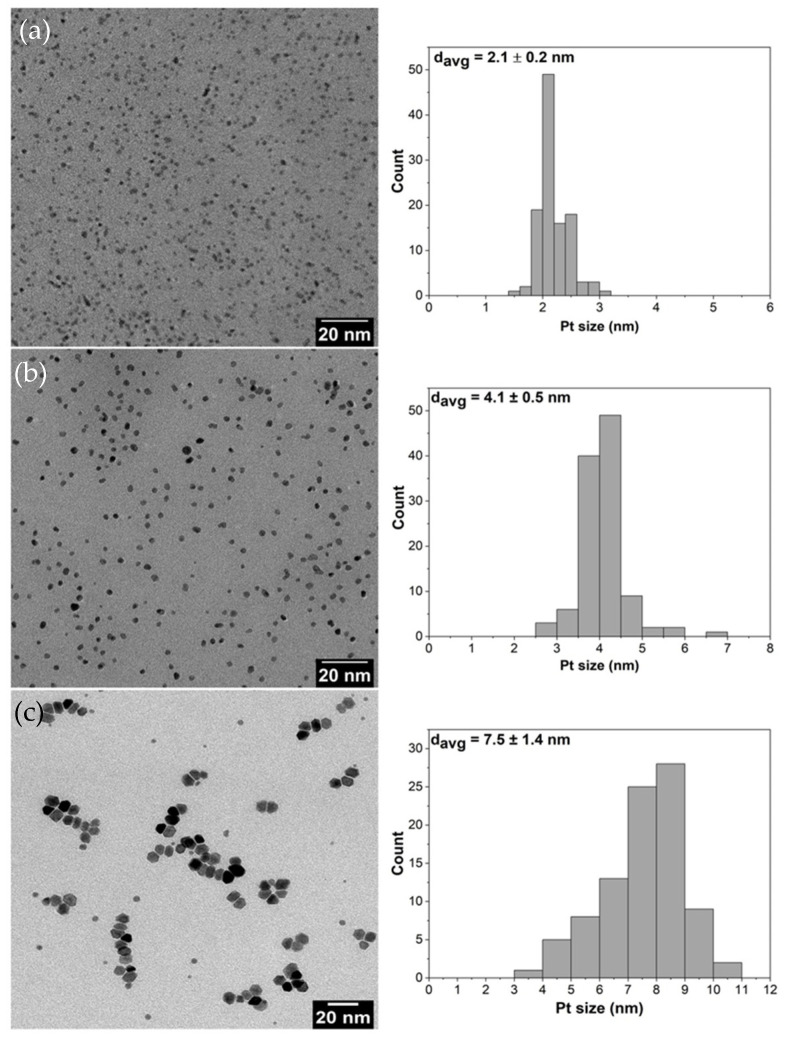
TEM images together with their particle size distributions of Pt nanoparticles with average sizes of: (**a**) 2 nm; (**b**) 4 nm; and (**c**) 8 nm.

Platinum (Pt) NPs with the average sizes of 2, 4 and 8 nm were synthesized using the polyol method described by Wang et al. [3]. Poly(vinylpyrrolidone) (PVP) was used as a capping agent and ethylene glycol as a solvent. For the 2 nm Pt NPs, 200 mg of NaOH, 160 mg of H_2_PtCl_6_ and 220 mg of PVP (Mw = 29,000) were dissolved into 20 mL of ethylene glycol and mixed together at 160 °C for 2 h under argon atmosphere. The resulting NPs were precipitated with 40 mL acetone and centrifuged. Hexane was used for washing repeatedly and the final product was dispersed in ethanol before use. For the 4 nm Pt NPs, 100 mg of H_2_PtCl_6_ and 440 mg of PVP (Mw = 29,000) were dissolved in 20 mL of ethylene glycol and mixed at 180 °C for 0.5 h under argon atmosphere. The resulting nanoparticles were precipitated with 40 mL acetone and centrifuged. Hexane was used for washing repeatedly and the final product was dispersed in ethanol before use. For the 8 nm Pt NPs, 160 mg of platinum (II) acetylacetonate and 220 mg of PVP (Mw = 29,000) were dissolved in 20 mL of ethylene glycol and mixed together at 200 °C for 2 h under argon atmosphere. The resulting nanoparticles were precipitated with 40 mL acetone and centrifuged. Hexane was used for washing repeatedly and the final product was dispersed in ethanol before use. Transmission electron microscopy (TEM) images of the prepared Pt NPs and their corresponding size distributions (Figure 1a–c) show that homogeneous morphologies as well as narrow size distributions were achieved during the preparation.

Two different types of silica, SBA-15 and MCF-17, were prepared according to the method reported in [2,29], respectively. In the preparation of SBA-15, 8 g of Pluronic were dissolved in 240 mL of 2 M HCl and 60 mL of water solution at 45 °C for 30 min and 17 g of TEOS were added to the solution with stirring at same temperature for 20 h. The resulting mixture was aged at 100 °C for 25 h. The white powder was recovered, washed with water and ethanol and calcined at 550 °C for 4 h to produce SBA-15. Preparation of MCF-17 was carried out by mixing 20 mL HCl, 130 mL water, 8 g of Pluronic and 8 g of TMB and stirring the mixture at 40 °C for 2 h. Then, 18 g of TEOS were added to the mixture, and, after stirring the mixture for 5 min, the solution was aged at 40 °C for 20 h. Next, 92 mg of NH_4_F were added and the solution was aged at 100 °C for 25 h. The precipitate product was filtered, washed with water and ethanol and calcined at 600 °C for 6 h. The white powder was collected as MCF-17 silica.

For the size effect study, synthesized Pt NPs were supported on two different types of silica (SiO_2_). The use of two types of silica support is attributed to different pore size requirements for Pt NPs loading on the internal surfaces of support [27]. Pt NPs with the sizes of 2 and 4 nm were loaded on SBA-15 with pore size ~7.4 nm, while 8 nm Pt NPs were loaded on MCF-17 silica with pore size ~40 nm. Loading of Pt NPs into silica support was carried out by sonication [30]. Calculated amounts of the colloidal solution of PVP-capped Pt NPs with targeted loading of 1.5 wt.% were mixed with 1 g of silica materials in a mixture (10 mL) 1:1 *v*/*v* of water and ethanol. The mixtures were sonicated for 3 h at room temperature. The precipitates were collected by a centrifuge (3000 rpm, 20 min) and dried at 80 °C for 12 h in oven.

### 2.2. Characterization

To analyze the shape, size and structure of the Pt NPs and Pt/SiO_2_ catalysts, transmission electron microscopy (TEM) analysis was performed with a JEM-2200 FS system operated at 200 kv. Samples were drop-casted on carbon-coated copper grids and subsequently air-dried before TEM analysis. The platinum contents of the prepared catalysts were analyzed after a microwave assisted sample digestion using an inductively coupled plasma optical emission spectrometer (ICP-OES, iCAP 6500 Duo, Thermo Fisher Scientific, Vantaa, Finland).

The specific surface area (S_BET_), net pore volume (V_net_) and pore-size distribution of catalysts were determined from nitrogen physisorption measurements performed using an ASAP2020 Micrometrics apparatus. As a pretreatment method before the physisorption measurements, the samples were degassed at 70 °C under 100 mmHg for 1440 min. Specific surface area, S_BET_ (m^2^ g^−1^), was calculated according to the classical Brunauer–Emmett–Teller (BET) theory for the p/p_0_ = (0.05–0.20). The pore-size distribution was evaluated from the adsorption branch of the nitrogen adsorption–desorption isotherm by the Barrett–Joyner–Halenda (BJH) method via the Roberts algorithm, using the Broekhoff–de Boer standard isotherm with the Faas correction and the assumption of the cylindrical-pore geometry (characterized by the diameter d_p_ of the pores). As the specific surface area (S_BET_) is not a proper parameter in the case of microporous-mesoporous solids [31], the mesopore surface area (S_meso_ in m^2^ g^−1^) and the micropore volume (V_micro_ in cm^3^_liq_ g^−1^) were also evaluated based on the t-plot method using the Broekhoff–de Boer standard isotherm. The net pore volume, V_net_ (cm^3^_liq_ g^−1^), was determined from the nitrogen adsorption isotherm at maximum p/p_0_~0.990.

X-ray photoelectron spectroscopy (XPS) surface analysis was conducted on Thermo Fisher Scientific ESCALAB 250Xi (East Grinstead, UK) with the settings: X-ray source: monochromatic Al Kα (1486.6 eV); X-ray power: 300 W; survey scan pass energy: 150 eV; and high-resolution scan pass energy: 20 eV. Separately, pristine Pt NPs and deposited Pt NP (Pt/SiO_2_) samples were dissolved in ethanol and dispersed on a gold sheet before the XPS measurement.

### 2.3. Activity Experiments

The activity experiments were conducted in an air-tight batch reactor, i.e., 250 mL three-neck round-bottom flask. In a typical experiment, 100 mL of synthetic solution of NaNO_3_ containing 100 mg/L of nitrates (${\mathrm{NO}}_{3}^{-}$) were purged by nitrogen gas at a flow rate of 100 mL/min for 20 min prior to the test, in order to remove the excess oxygen. Aqueous solution of (0.01 mM) NaBH_4_ was prepared in presence of sodium hydroxide (0.2%) and then 0.1 g of catalyst in a powder form was reduced by adding dropwise followed by vacuum-filtration [32]. The reduced catalyst was washed with Milli-Q water twice to remove residual chemicals and immediately transferred to the reactor. Hydrogen gas was introduced into the catalyst solution through a sintered glass tube at the flow rate of 100 mL/min to serve as a reductant. The reactor was placed in a water bath and kept at constant temperature (25 °C), and the reaction mixture was continuously mixed with a Teflon coated magnetic stir-bar at 400 rpm. To verify the results, second cycle activity experiment was carried out with the same filtered and dried (overnight in 50 °C) 4 nm Pt/SiO_2_ catalyst. This test showed that the catalyst remained active during the tests.

Water sample analyses during the experiments were done every 10 min by an online capillary electrophoresis (CE) Sciex P/ACE MDQ Plus system (Berner Pro, Helsinki, Finland). Capillary electrophoresis analytical technique separates ions based on their electrophoretic mobility with the use of an applied voltage [33,34]. The measured species in water samples were nitrate (${\mathrm{NO}}_{3}^{-}$), nitrite (${\mathrm{NO}}_{2}^{-}$) and ammonium (${\mathrm{NH}}_{4}^{+}$) concentrations. The reactor was connected to the CE system via an on-line functioning apparatus, and it was programmed to introduce a sample into the instrument every 10 min. The concentration of nitrate and nitrite was determined by using CEofix™ Anions2 kit with anion buffers and a 75 µm × 50 cm fused silica capillary (Berner Pro, Helsinki, Finland). Samples were introduced into the capillary by pressure injection. A 10 kV power supply with a reversible polarity output was employed as a source for the separation. Nitrate and nitrite were separated below 5 min migration time and detection was achieved with a diode array detector (DAD) at 214 nm. After each test, the final concentration of ammonium ions was determined by a CE system by using CEofix™ Cations HR kit with cation buffers and a 75 µm × 50 cm fused silica capillary (Berner Pro, Helsinki, Finland). A 30 kV voltage power supply with a normal polarity output was employed as a source for the separation. The detection of the sample was achieved with a diode array detector (DAD) at 200 nm. By assuming that only negligible amounts of other intermediate products were formed (e.g., NO  and $N_{2}O$) [32], the selectivity to nitrogen ($S_{N_{2}}$) was calculated as follows:

(1) $$S_{N_{2}}=100\%-S_{{\mathrm{NH}}_{4}^{+}}-S_{{\mathrm{NO}}_{2}^{-}}$$

where the selectivity to ammonium ($S_{{\mathrm{NH}}_{4}^{+}}$) and nitrite ($S_{{\mathrm{NO}}_{2}^{-}}$) are based on the measured ${\mathrm{NH}}_{4}^{+}$ and ${\mathrm{NO}}_{2}^{-}$ concentrations and calculated as follows:

(2) $$S_{{\mathrm{NH}}_{4}^{+}}=\left(\frac{C_{{\mathrm{NH}}_{4}^{+}}}{C_{0}-C_{f}}\right)\times 100,$$

and

(3) $$S_{{\mathrm{NO}}_{2}^{-}}=\left(\frac{C_{{\mathrm{NO}}_{2}^{-}}}{C_{0}-C_{f}}\right)\times 100,$$

with $C_{0}$ the measured initial concentration and $C_{f}$ the measured final concentration of nitrate (${\mathrm{NO}}_{3}^{-}$). The NPs size effect on activity was evaluated by calculating TOF values for each catalyst. The turnover frequency (TOF) values are based on initial rate of reaction (first 20 min) and number of active sites were calculated based on TEM (particle size) and ICP-AES (Pt loading data) data, assuming spherical shape of Pt nanoparticles [3]. Since inert silica was used as a support in all three catalysts, reactions were assumed to have taken place on the surface of Pt metal, assuming that all the surface atoms were available and took part in reduction reaction [35].

## 3. Results and Discussion

### 3.1. Characterization of the Prepared Catalysts

**Figure 2:**
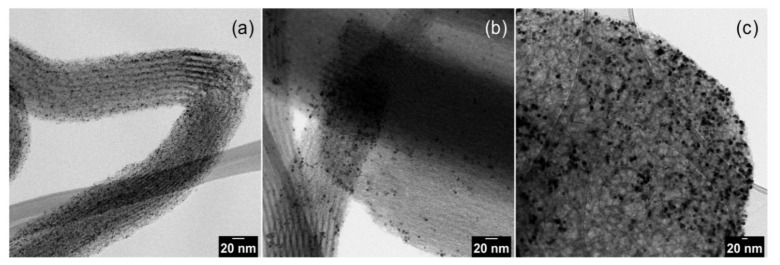
TEM images of Pt nanoparticles dispersed on silica supports: (**a**) 2 nm Pt on SBA-15; (**b**) 4 nm Pt on SBA-15; and (**c**) 8 nm Pt on MCF-17.

The TEM images of pristine Pt NPs are shown in Figure 1 and Pt NPs dispersed on silica supports are presented in Figure 2. The obtained nanoparticles showed well-controlled average diameters with narrow size distributions (Figure 1). Figure 2 shows that incorporation of Pt NPs in silica support (SBA-15 and MCF-17) was successfully achieved by the sonication method. Sonication can prevent blockage of the pores by helping the Pt particles to diffuse into the channels, and surface tension of the inclusion solvent (water/ethanol mixture) is an important parameter for successful deposition of Pt NPs into mesoporous supports [2]. Pt NPs with sizes 2 and 4 nm (Figure 2a,b) are well-dispersed in the entire channel structures of silica (SBA-15), while some aggregation of 8 nm Pt NPs is visible (Figure 2c) on the surface of MCF-17.

**Figure 3:**
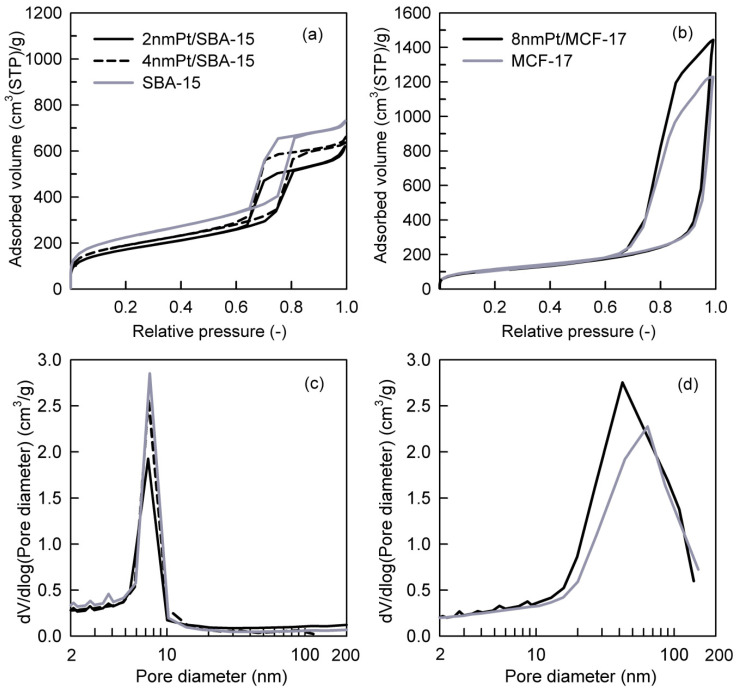
Nitrogen adsorption–desorption isotherms of (**a**) SBA-15-based and (**b**) MCF-17-based catalysts and evaluated pore-size distributions of (**c**) SBA-15-based and (**d**) MCF-17-based catalysts.

Pt loadings in the catalysts and their textural properties are summarized in Table 1. Platinum loading (wt.%) varied from 1.2% to 1.8%, and it was taken into consideration while calculating the TOF values; therefore, comparative difference in Pt loading can be compensated [36]. The measured nitrogen adsorption–desorption isotherms and the pore-size distributions (PSDs) of investigated catalysts are shown in Figure 3a–d. The shape of hysteresis loops of nitrogen adsorption–desorption isotherms of SBA-15 silica-based catalysts (Figure 3a) corresponds to the H1 loop type which proves the uniform narrow cylindrical mesopores with a diameter of ~7.4 nm in the Pt NPs loaded catalysts as well as the SBA-15 support (Figure 3c). When comparing 2 nm Pt/SBA-15 and 4 nm Pt/SBA-15 catalysts with the pristine SBA-15 support, it is evident that the different size of deposited Pt NPs on the SBA-15 support did not affect the pore size of the Pt NPs loaded catalysts (Figure 3c). This fact indicates that Pt NPs are deposited freely on the internal and external surfaces of mesopores and do not aggregate significantly. This observation is in a nice agreement with the TEM images where it is obvious that 2 and 4 nm Pt NPs are well-dispersed on the SBA-15 support (Figure 2a,b) and as-synthesized PVP-capped Pt NPs do not aggregate (Figure 1a,b). The decrease in the mesopore surface area and the net pore volume of the Pt NPs loaded catalysts compared to the pristine SBA-15 support is caused by the presence of Pt NPs. The shape and width of the hysteresis loop of the nitrogen adsorption–desorption isotherms concerning 8 nm Pt/MCF-17 and the MCF-17 silica support (Figure 3b) is of the H2b loop type, revealing the existence of more complex porous structure with a large range of pore sizes in both, which is reflected to their similarly broad pore-size distributions (Figure 3d). However, while 8 nm Pt/MCF-17 shows PSD with the pore diameter maximum at ~40 nm (i.e., the most frequent pores of 40 nm diameter are present), the MCF-17 support shows PSD with the pore diameter maximum at ~65 nm. This feature indicates that 8 nm Pt NPs are probably more aggregated, create NPs clusters in MCF-17 porous structure which led to the decrease in the pore size. A similar phenomenon was also detected with the TEM analyses when the higher tendency of as-synthesized 8 nm PVP-capped Pt NPs to aggregate was recognized (Figure 1c) and aggregates of 8 nm Pt NPs loaded on MCF-17 were seen (Figure 2c).

### 3.2. Influence of Pt Nanoparticle Size on Activity in Nitrate Reduction

**Figure 4:**
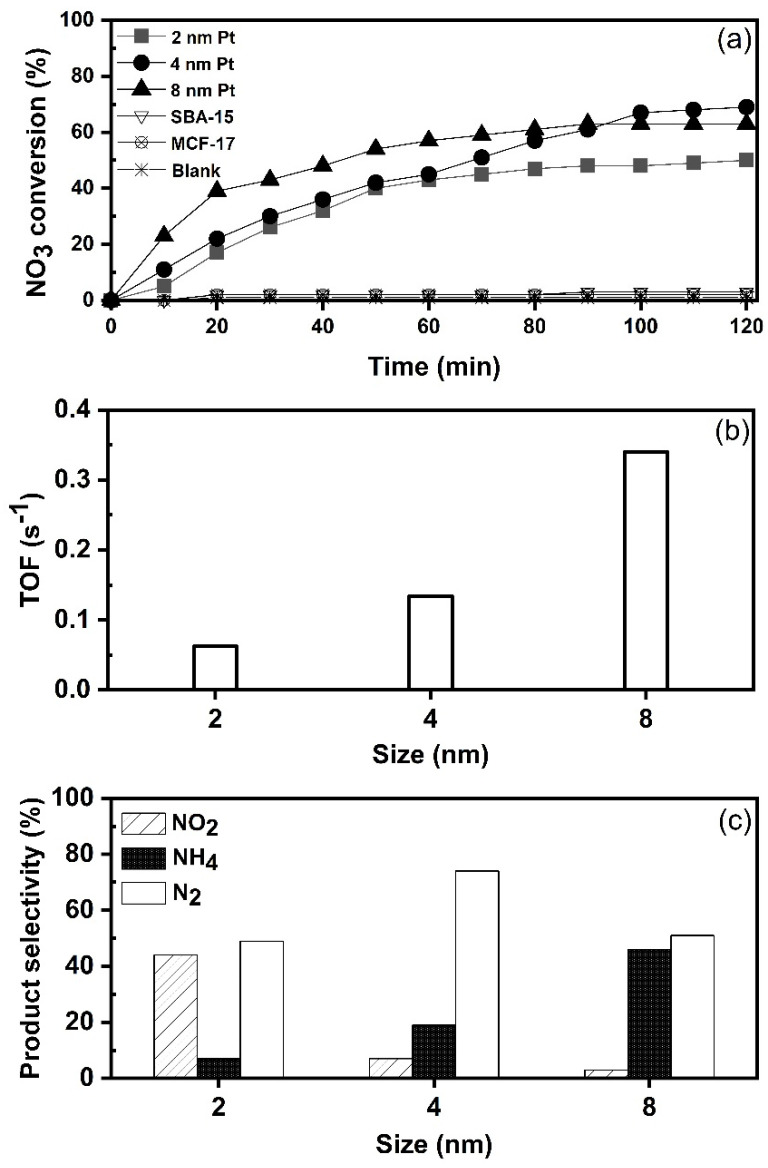
Effect of Pt nanoparticle size on nitrate reduction (catalyst 1 g L^−1^, ${\mathrm{NO}}_{3}^{-}$ 100 mg L^−1^, $H_{2}$ 100 mL min^−1^ at 25 °C): (**a**) nitrate conversion with time; (**b**) TOF values calculated at first 20 min reaction time (mM of ${\mathrm{NO}}_{3}^{-}$ molecules converted per active site per s); and (**c**) product selectivity at 120 min of reaction time.

At the beginning of the study, activities of mesoporous silica (SBA-15 and MCF-17) were evaluated for nitrate reduction. Figure 4a shows that nitrate (${\mathrm{NO}}_{3}^{-}$) cannot be directly reduced by the support itself (SBA-15 and MCF-17), or by hydrogen gas (H_2_), without a catalyst in a blank test. This indicates that nitrate (${\mathrm{NO}}_{3}^{-}$) reduction process occurred only on supported Pt NPs. In the next step, catalysts with the average Pt size of 2, 4 and 8 nm for nitrate (${\mathrm{NO}}_{3}^{-}$) hydrogenation were tested at ambient conditions. The turnover frequency (TOF) values shown in Figure 4b were calculated in the first 20 min based on the initial rate and correspond to mM of molecules converted per active site per second. The results reveal the dependency of catalytic activity on the Pt nanoparticle size. The TOF value of nitrate reduction is higher with 8 nm Pt than with 4 and 2 nm Pt size. The size-dependent activity suggests that large Pt NPs are more active in nitrate hydrogenation in the size range of 2–8 nm. As suggested by Choi et al. [37], large Pt NPs consist mostly of low coordination sites (including edges and steps) where reaction substrate could be adsorbed more strongly. Therefore, the TOF value of 0.34 s^−1^ for 8 nm is decreased to 0.13 and 0.06 s^−1^ for 4 and 2 nm Pt NPs, respectively. Overall, the nitrate reduction reaction appeared to be clearly dependent on the size of Pt NPs and the trend was monotonic in the size range from 2 to 8 nm. Similar results can be found in previous reports which used Pd on various supports [38,39]. At 120 min of reaction time, nitrate conversions (Figure 4a) reached 50%, 63% and 70% over the 2, 8 and 4 nm Pt, respectively. After 100 min, the conversions were nearly stabilized, i.e., none of the catalysts achieved complete nitrate removal from the reaction mixture. This is most likely attributed to the change in pH as the reaction proceeds with time, since the catalysts were tested without controlling the pH of reaction mixture. For example, with 4 nm Pt/SiO_2_ catalyst, the pH changed from 6.7 to 6.85 during the first 20 min, reaching 7.2 at 60 min, 7.8 at 100 min and 8.1 at 120 min. A gradual decrease in the reduction reaction is due to accumulation of hydroxide ions (${\mathrm{OH}}^{-}$) [40,41]. It is also possible that some degree of aggregation of nanoparticles exists in catalysts [3], which could could affect the activity as well.

According to the results (Figure 4c), nitrates are transferred to different products such as nitrogen ($N_{2}$), nitrite (${\mathrm{NO}}_{2}^{-}$) and ammonium (${\mathrm{NH}}_{4}^{+}$) on all three catalysts. However, all three catalysts showed different selectivity. The product distributions (%) after 120 min of reaction time, as shown in Figure 4c, were as follows: 2 nm Pt produced 44% ${\mathrm{NO}}_{2}^{-}$, 7% ${\mathrm{NH}}_{4}^{+}$ and 49% $N_{2}$, from the overall conversion of nitrate. The 4 nm Pt shows a higher selectivity (74%) towards $N_{2}$, with only small amount of ${\mathrm{NO}}_{2}^{-}$ (7%) formed, and selectivity towards ${\mathrm{NH}}_{4}^{+}$ was 19%. The 8 nm Pt showed high selectivity toward ${\mathrm{NH}}_{4}^{+}$ (46%) and only small amount of ${\mathrm{NO}}_{2}^{-}$ was detected. Based on the results, it can be suggested that Pt NPs with the size of 2 nm become too small to adsorb and activate two N species for the formation of nitrogen ($N_{2}$). A similar observation was reported by Zhang et al. [42] in the case of palladium as the active metal. In our study, platinum particles with an average size of 4 nm were the most selective towards the formation of nitrogen ($N_{2}$). On the other hand, the selectivity towards ammonium (${\mathrm{NH}}_{4}^{+}$) is higher over 8 nm Pt NPs when compared to 2 and 4 nm NPs. The size-dependent selectivity suggests that larger Pt NPs are more active for ${\mathrm{NH}}_{4}^{+}$ formation. As mentioned by Dong et al. [43], when the size of Pt NPs increases, the proportion of terrace sites also increases. For example, according to Lundwall et al. [44], the amount of terrace sites increases by 50% when the Pt particle size is increased from 2.5 to 4.2 nm. The correlation between the selectivity for ${\mathrm{NH}}_{4}^{+}$ and size of Pt NPs suggests that terrace sites of Pt NPs are the active sites for ${\mathrm{NH}}_{4}^{+}$ formation.

It is noteworthy that, in previously reported studies [45,46,47], monometallic catalysts were found to be inactive for nitrate (${\mathrm{NO}}_{3}^{-}$) reduction and addition of co-metals, such as In, Cu and Sn are typically required to reduce the nitrate in an aqueous phase. However, Huo et al. [48] showed that reduction of aqueous nitrate (${\mathrm{NO}}_{3}^{-})$ was possible by using monometallic Ru/C catalyst at controlled pH condition (pH 5.0). They showed that the activity of Ru/C catalyst was due to pretreatment of the catalyst with H_2_ gas at 350 °C for 2 h. Nevertheless, for Ru/C catalyst, nitrate was converted to ammonium (${\mathrm{NH}}_{4}^{+})$ without conversion to harmless nitrogen (N_2_) gas. Similar results were shown by Soares et al. [21], who used Ru catalyst supported on activated carbon (AC) and almost 15 ppm of nitrate (${\mathrm{NO}}_{3}^{-})$ converted in 300 min of reaction time. Moreover, Epron et al. [49] reported the catalytic reduction of nitrate (${\mathrm{NO}}_{3}^{-})$ by using Pd/CeO_2_ catalyst and highlighted the promoting effect of ceria support based on its redox properties. Several studies have documented that activity of supported metal nanoparticles is influenced by nanoparticle size, metal–support interaction, synthesis method and capping agent [50,51,52].

**Figure 5:**
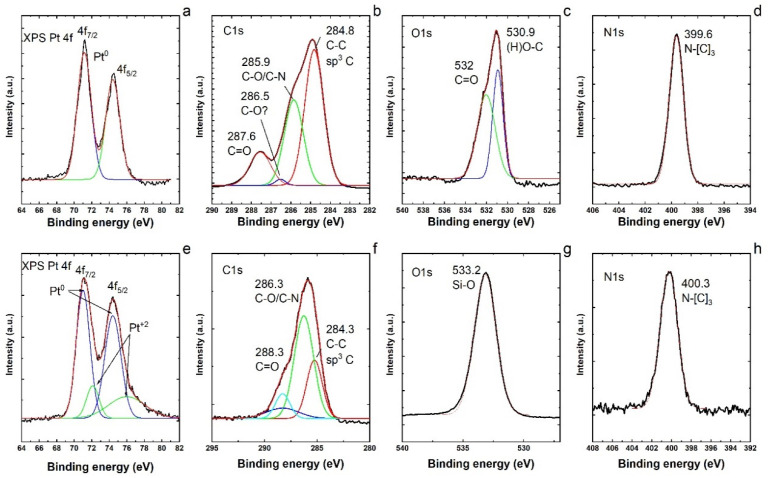
(**a**) XPS Pt 4f; (**b**) C1s; (**c**) O1s; and (**d**) N1s regions of XPS spectra of Pt nanoparticles before the loading on the silica support. (**e**) XPS Pt 4f; (**f**) C1s; (**g**) O1s; and (**h**) N1s regions of the XPS spectra of Pt nanoparticles after the loading on the silica support.

Therefore, to gain more understanding on the nitrate reduction reaction with a monometallic catalyst (Pt/SiO_2_) in our study, XPS analyses of Pt NPs before and after the loading of Pt on the silica (SiO_2_) support were conducted, as presented in Figure 5. The XPS spectra of both samples exhibited the presence of C, O and N species apart from Pt [53]. The XPS Pt 4f (Figure 5a) shows that Pt is in the metallic form Pt^0^ [54]. The peaks look very symmetric and no assignation to Pt^+1^ or Pt^+2^ could be made. Analyzing the C1s spectrum (Figure 5b), it is possible to observe signals at 248.8 (sp^3^ C–C), 285.9 (C–N/C–O) and 287.6 (C=O). It is also possible to distinguish a small signal at 286.5, tentatively assigned to (C–O). The O1s spectrum (Figure 5c) shows signals at 530.9 and 532 assigned to (H)O–C and C=O. The N1s spectrum (Figure 5d) shows only one signal at 399.6 assigned to N–[C]_3_, that is, the nitrogen atom bonded to three carbon atoms [55]. The XPS Pt 4f (Figure 5e) shows two peaks that are not symmetric. These peaks were fitted with two Gaussians each and are assigned to Pt^0^ and Pt^+2^ (PtO). However, most of the Pt is in the metallic form as Pt^0^. These peaks seem broader than the peaks in Figure 5a. Probably, Pt is attached to the SiO_2_ surface by Pt–O interactions. Analyzing the C1s spectrum (Figure 5f), it is possible to observe signals at 248.3 (sp^3^ C–C), 286.3 (C–N/C–O) and 288.3 (C=O). The ratio of (C–N/C–O)/(C–C) is higher and the signal for C=O is lower. This could indicate that the PVP molecule is interacting more with the substrate SiO_2_ via the O atom. The O1s spectrum (Figure 5g) shows a major signal that governs the spectrum at 533 assigned to the Si–O bonding of SiO_2_. The N1s spectrum (Figure 5h) shows only one signal at 400.3 assigned to N–[C]_3_, that is, the nitrogen atom bonded to three carbon atoms. There is a small shift in the N–[C]_3_ signal that could indicate some variations in the PVP ligands. By comparing the identified elements and bonding, we conclude that the PVP molecules are partially modified after the loading of Pt NPs on the silica support.

The N1s XPS signals show the appearance of nitrogen species (Figure 5d,h) which can only come from the capping agent such as PVP [56]. Chemisorbed PVP ligands were reported to enhance the catalytic activity in the liquid phase reaction by stabilizing the metal nanocrystals [57]. The pyrrolidone ring of chemisorbed PVP interacts with the Pt surface and that interaction can serve as the electron donor–acceptor media between PVP and the Pt surface [58]. However, the interaction of PVP ligands with the metal via the O atom in the ring can accompany the charge transfer from PVP to the metal, and the PVP interaction via the N atom in the ring can accompany the charge transfer from the metal to PVP [59]. It has also been reported [60] that the nanoparticle size can be a determining factor in the PVP-stabilized metal surface, serving either as an electron donor or as an electron acceptor. We therefore conceive that this distinct electronic structure property of Pt–PVP NPs is resulting in higher activity of a monometallic catalyst in our case, and the PVP ligands apparently work as a promoter in the PVP capped Pt NPs.

## 4. Conclusions

The size effect of Pt nanoparticles (NPs) supported on silica in catalytic reduction of nitrates in terms of activity and selectivity was studied. Under the reaction conditions used in this study, the Pt NPs size had a noticeable effect on the TOF values of nitrate reduction, ascending in the size range of 2–8 nm. Nitrogen was a relatively dominant product in all studied catalysts, but 4 nm Pt showed the best selectivity. The size-dependent selectivity showed that large Pt NPs favored ammonium formation, which can be associated with increased proportional terrace sites being an active ground for the ammonium ions formation. In contrast, the smaller 2 nm Pt NPs were found to be too small for nitrogen formation. The role of the PVP ligands on the Pt NP surfaces was also discussed. Besides the Pt NP size effect, the interaction of the capping agent with metal particles could be regarded as an important aspect for the catalytic behavior of size-controlled nanoparticles. The next step towards developing new technology for catalytic reduction of nitrogen compounds in wastewater treatment is to study the effect of platinum interactions with different supports and the effect of different reaction conditions.

## Figures and Tables

**Table 1:** Textural properties and Pt loadings of SBA-15 and MCF-17 supported catalysts.

Catalyst	Pt Loading (wt.%)	S_BET_ (m^2^g^−1^)	S_meso_ (m^2^g^−1^)	V_micro_ (cm^3^_liq_g^−1^)	V_net_ (cm^3^_liq_g^−1^)
SBA-15	-	799	714	0.03	1.11
2 nm Pt/SBA-15	1.5	615	565	0.01	0.93
4 nm Pt/SBA-15	1.2	683	587	0.03	0.98
MCF-17	-	421	421	0	1.90
8 nm Pt/MCF-17	1.8	397	397	0	2.23

## Data Availability

The data presented in this study are available on request from the corresponding author. All results have been published in this article and the original data (notebooks, raw data files from experiments and characterization) have been self-archived according to the instructions given by University of Oulu.
